# The Prediction of Membrane Protein Structure and Genome Structural Annotation

**DOI:** 10.1002/cfg.308

**Published:** 2003-07

**Authors:** Pier Luigi Martelli, Piero Fariselli, Gianluca Tasco, Rita Casadio

**Affiliations:** Laboratory of Biocomputing CIRB/Department of Biology University of Bologna via Irnerio 42 Bologna 40126 Italy

## Abstract

New methods, essentially based on hidden Markov models (HMM) and neural networks (NN), can predict the topography of both β-barrel and all-α membrane proteins with high accuracy and a low rate of false positives and false negatives. These methods have been integrated in a suite of programs to filter proteomes of Gram-negative bacteria, searching for new membrane proteins.


**Comparative and Functional Genomics**
*Comp Funct Genom* 2003; **4**: 406–409.
Published online in Wiley InterScience (www.interscience.wiley.com). **DOI:** 10.1002/cfg.308

**Conference Review**

### The prediction of membrane protein structure and genome structural annotation

Pier Luigi Martelli, Piero Fariselli, Gianluca Tasco and Rita Casadio*
*Laboratory of Biocomputing, CIRB/Department of Biology, University of Bologna, via Irnerio 42, 40126 Bologna, Italy*

*Correspondence to:
Rita Casadio, Laboratory of Biocomputing, CIRB/Department of Biology, University of Bologna, via Irnerio 42, 40126 Bologna, Italy.
E-mail: {gigi/piero/gluca}@biocomp.unibo.it; casadio@alma.unibo.it

Received: 3 June 2003
Revised: 3 June 2003
Accepted: 3 June 2003

### Abstract

**New methods, essentially based on hidden Markov models (HMM) and neural networks (NN), can predict the topography of both $\beta$-barrel and all-$\alpha$ membrane proteins with high accuracy and a low rate of false positives and false negatives. These methods have been integrated in a suite of programs to filter proteomes of Gram-negative bacteria, searching for new membrane proteins. Copyright © 2003 John Wiley & Sons, Ltd.**

**Keywords:** **membrane protein prediction; neural network; hidden Markov models; genome structural annotation**

### Introduction

Presently less than 1% of the PDB structures are membrane proteins solved with atomic resolution. Considering also that membrane proteins, even when they belong to the same functional family, share little sequence identity, it is still problematic to develop low-resolution models, with very few exceptions. For this reason, efforts have been directed towards the high accuracy prediction of the location of transmembrane segments along protein sequences (topography) as well as the positions of the N- and C-termini of proteins, with respect to the membrane plane [3]. These predictors take advantage of the fact that membrane proteins are constrained by lipid bilayers of different composition to just two known types of architecture: the so-called all-$\alpha$ helical proteins of the inner membrane, and the $\beta$-barrel proteins of the outer membrane of Gram-negative bacteria.

Among all the possible methods described to predict the topology of all-$\alpha$ membrane proteins [3], two are particularly interesting, and consider two different approaches.

Transmembrane propensity scales indicate, on the basis of physicochemical properties, the membrane interacting segments along a protein chain. Experiment-based scales (MPXe) make it possible to account, for example, for the effect of buried salt bridges on hydropathy plot results, which is extremely important when considering proteins involved in ion transport (contributed by Stephen White [9,10]), and, used in combination with other scales (MPtopo) can also predict membrane protein topology.

Alternatively, prediction methods such as TMHMM and HMMTOP [11,16], based on automatic learning, can identify the helix bundle membrane proteins encoded in fully sequenced genomes with very high precision (sensitivity and specificity both around 95%). Topology prediction is also quite good (correct predictions 65–70% of the time). Recently, new topologies have been determined by a combination of topology predictions and standard experimental approaches such as PhoA- or GFP-fusions [5] (contributed by Gunnar von Heijne).

We developed different predictors for the all-$\beta$ and one for the all-$\alpha$ membrane proteins,

Copyright © 2003 John Wiley & Sons, Ltd.

respectively [8,13]. Considering that these predictors are presently among the best performing of their type [3], we propose to integrate them into a suite of predictors (Hunter) capable of filtering the whole proteome for finding new membrane proteins [1]. By this, it is proposed that structural genomics tools can help in classifying ORFs into structural classes.

### Prediction of all-$\beta$ membrane proteins

The outer membrane of Gram-negative bacteria contains unique membrane proteins folded into $\beta$-barrel-like structures, comprising an even number of antiparallel $\beta$-strands. This type of architecture is well conserved among prokaryotes [15]. Recently, it has also been proposed that in the outer membrane of chloroplasts and mitochondria, proteins containing a similar membrane interacting portion can perform different functions, including voltage-dependent anion channel (VDAC) [2] and unfolded protein transport (TOM40) [7]. To date, the development of methods suited to locating the membrane-spanning regions of these proteins, starting from the sequence, has been scarce [18].

We have implemented two different methods, both based on machine learning, to predict the topography of this type of membrane protein. The first (IRENE, **http://www.biocomp.unibo.it**, [8]) is based on neural networks, and the second is based on HMMs (CINZIA [13]). The two methods, trained and tested with a jackknife procedure on sequence profiles, and endowed with a dynamic programming-based filter [6], perform similarly on a set of 15 non-redundant all-$\beta$ membrane proteins taken from the PDB. The HMM-based method is superior to NN only when both are trained with sequence profiles [13].

However, when tested on a much larger set of well-annotated sequences from the SWISS PROT database, the rate of false positives is lower in the case of the HMM-based predictor. This is due to the intrinsic capability of HMMs to act as more stringent filters than NN, especially when, as in this case, patterns are rather difficult to discriminate based only on the local context. Indeed, transmembrane $\beta$-strands are rather similar to the $\beta$-strands of globular proteins in both composition and pattern [8].

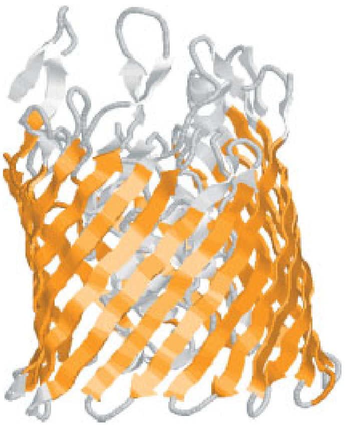

| Expected | Predicted |
|---|---|
| 136-146 | 140-146 |
| 148-157 | 152-159 |
| 165-176 | 164-175 |
| 196-211 | 199-211 |
| 214-228 | 214-225 |
| 242-257 | 244-257 |
| 261-276 | 261-273 |
| 290-305 | 292-305 |
| 309-322 | 309-320 |
| 332-348 | 335-347 |
| 351-361 | 351-361 |
| 366-378 | 369-378 |
| 383-395 | 383-394 |
| 413-425 | 414-425 |
| 430-441 | 429-441 |
| 458-470 | 459-471 |
| 477-487 | 475-486 |
| 500-510 | 500-510 |
| 515-524 | 514-527 |
| 542-553 | 541-554 |
| 558-566 | 559-568 |
| 584-594 | 584-594 |

**Figure 1.** Prediction the transmembrane domain of the outer membrane cobalamin transporter from *Escherichia coli* (1nqf) with CINZIA. The protein was not included in the training set

Starting from the sequence of any outer membrane protein of mitochondria and chloroplasts, it is possible to locate the transmembrane strands along the chain (Figure 1). If the number of strands is equal to that of a known $\beta$-barrel in the database, then threading is possible. The alignment can be made with the constraint that the positions of the $\beta$-strands are conserved. This procedure made possible the evaluation of the first model of a voltage-dependent anion channel capable of fitting most of the experimental data published so far [2].

### Prediction of all-$\alpha$ membrane proteins

Membrane proteins in the inner membrane fold as $\alpha$-helix bundles [17]. Several predictors are available through the web [3,4]. However, none of them have been specifically trained on the structures available with atomic resolution. Prompted by the finding that most predictors fail in correctly locating the positions of the membrane spanning regions of recently crystallized proteins, we implemented a tool integrating one neural network and two HMMs, suited to predicting more and less hydrophobic helices (ENSEMBLE) [12]. The predictor trained and tested with the sequence profiles of 59 chains of well-resolved membrane proteins with a jackknife procedure, correctly predicts the topography of 53 proteins (Figure 2).

Copyright © 2003 John Wiley & Sons, Ltd. 

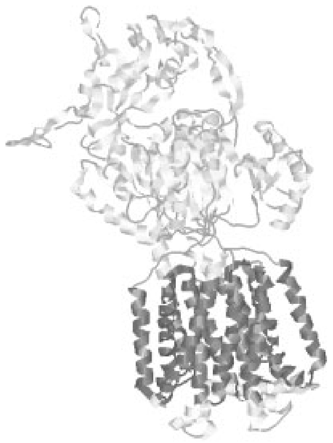

| Expected | Predicted |
|---|---|
| 8-28 | 9-28 |
| 329-357 | 339-360 |
| 361-385 | 362-388 |
| 391-423 | 390-417 |
| 437-458 | 436-459 |
| 463-496 | 468-497 |
| 538-558 | 534-558 |
| 872-892 | 872-893 |
| 896-919 | 895-921 |
| 924-956 | 923-950 |
| 972-991 | 969-991 |
| 997-1033 | 1003-1030 |

**Figure 2.** Prediction of the transmembrane regions of the multidrug efflux transporter AcrBn from *Escherichia coli* (1iwg) with ENSEMBLE. The protein was not included in the training set. The predicted transmembrane regions are shown as dark regions on the protein ribbon diagram

### Proteome filtering

A requisite of any predictor used to screen a whole proteome is that the rate of false predictions is known. An estimate of this measure can be obtained by filtering some 1000 non-redundant chains including different structural classes of globular proteins, and a large set of well-annotated chains of membrane proteins from the SWISS PROT database.

The reported rate of false predictions for both CINZIA [13] and ENSEMBLE [12] is rather low (10% of false positives and 15% of false negatives for CINZIA, 3% of false positives and 3% of false negatives for ENSEMBLE, respectively). A possible application of these predictors is therefore genome screening, with the specific aim of fishing out new membrane proteins. This issue has so far been addressed separately in the literature for either type of membrane proteins. In our case, we implemented a suite of predictors (HUNTER, [1]) that also includes a signal peptide predictor of the SignalP type [14], and is capable of screening a proteome of some 5000 proteins in about 2 days, on a 1GHz PC.

The results of this analysis, as performed on a selection of freely available genomes of Gram-negative bacteria, suggests that the approximate content of alpha helical membrane proteins is in the range of 20%, confirming the results of other similar screenings with different predictors. However, the novelty is that the amount of beta barrel membrane proteins in the proteome is in the range of 1–2% of the whole protein content and that this is quite independent of the type of bacterium, and of its pathogenicity. In this way, complete lists of proteins in a given structural class (membrane all-$\alpha$, or all-$\beta$, and globular, as a complement of the first two types) can be produced and can then be aligned to the SWISS PROT database. Using this procedure, those ORFs that have not yet been assigned an annotation, that are classified in either class of membrane proteins, can be highlighted. The non-annotated protein list is then available for experimental determination. We suggest that this tool can help in focusing on those proteins of the proteome that still lack annotation.

### Acknowledgements

This work was partially supported by grants from the Ministero della Università e della Ricerca Scientifica e Tecnologica (MURST) for the project "Hydrolases from thermophiles: Structure, function and homologous and heterologous expression" and for the project "Development and implementation of algorithms for predicting protein structure", and from the Italian Centro Nazionale delle Ricerche (CNR) for projects on Molecular Genetics, and Functional Genomics, and also by a PNR 2001–2003 (FIRB art.8) project on Postgenomics (all awarded to RC). PLM was the recipient of a fellowship from the Italian Center for National Researches (CNR) devoted to a target project on Molecular Genetics (Law No 449-1997).

### References

1. Casadio R, Fariselli P, Finocchiaro G, Martelli PL. 2003. Fishing new proteins in the twilight zone of genomes: the test case of outer membrane proteins in *Escherichia coli* K12, *Escherichia coli* O157:H7, and other Gram-negative bacteria. *Protein Sci* **2**: 1158–1168.
2. Casadio R, Jacoboni I, Messina A, De Pinto V. 2002. A 3D model of the voltage-dependent anion channel (VDAC). *FEBS Lett* **520**: 1–7.
3. Chen CP, Kernytsky A, Rost B. 2002. Transmembrane helix predictions revisited. *Protein Sci* **11**: 2774–2791.
4. Chen CP, Rost B. 2002. Long membrane helices and short loops predicted less accurately. *Protein Sci* **11**: 2766–2773.
5. Drew D, Sjostrand D, Nilsson J, *et al.* 2002. Rapid topology mapping of *Escherichia coli* inner-membrane proteins by prediction and PhoA/GFP fusion analysis. *Proc Natl Acad Sci USA* **99**: 2690–2695.
6. Fariselli P, Finelli M, Marchignoli D, *et al.* 2003. MaxSubSeq: an algorithm for segment-length optimization. The case study of the transmembrane spanning segments. *Bioinformatics* **19**: 500–505.

Copyright © 2003 John Wiley & Sons, Ltd. 

7. Gabriel K, Egan B, Lithgow T. 2003. Tom40, the import channel of the mitochondrial outer membrane, plays an active role in sorting imported proteins. *EMBO J* **22**: 2380–2386.
8. Jacoboni I, Martelli PL, Fariselli P, De Pinto V, Casadio R. 2001. Prediction of the transmembrane regions of $\beta$-barrel membrane proteins with a neural network-based predictor. *Protein Sci* **10**: 779–787.
9. Jayasinghe S, Hristova K, White SH. 2001. Energetics, stability, and prediction of transmembrane helices. *J Mol Biol* **312**: 927–934.
10. Jayasinghe S, Hristova K, White SH. 2001. MPtopo: a database of membrane protein topology. *Protein Sci* **10**: 455–458.
11. Krogh A, Larsson B, von Heijne G, Sonnhammer EL. 2001. Predicting transmembrane protein topology with a hidden Markov model: application to complete genomes. *J Mol Biol* **305**: 567–580.
12. Martelli PL, Fariselli P, Casadio R. 2003. An ENSEMBLE machine learning approach for the prediction of all-$\alpha$ membrane proteins. *Bioinformatics* (in press).
13. Martelli PL, Fariselli P, Krogh A, Casadio R. 2002. A sequence-profile-based HMM for predicting and discriminating $\beta$-barrel membrane proteins. *Bioinformatics* **18**(suppl 1): S46–53.
14. Nielsen H, Brunak S, von Heijne G. 1999. Machine learning approaches to the prediction of signal peptides and other protein sorting signals. *Protein Eng* **12**: 3–9.
15. Schulz GE. 2003. Transmembrane $\beta$-barrel proteins. *Adv Protein Chem* **63**: 47–70.
16. Tusnady GE, Simon I. 1998. Principles governing amino acid composition of integral membrane proteins: application to topology prediction. *J Mol Biol* **283**: 489–506.
17. von Heijne G. 1999. Recent advances in the understanding of membrane protein assembly and structure. *Q Rev Biophys* **32**: 285–307.
18. Wimley WC. 2002. Toward genomic identification of $\beta$-barrel membrane proteins: composition and architecture of known structures. *Protein Sci* **11**: 301–312.

Copyright © 2003 John Wiley & Sons, Ltd. 
